# The Role of Exogenous Non-Starch Polysaccharide Enzymes in Enhancing Digestibility and Performance of Pig

**DOI:** 10.3390/biology15010013

**Published:** 2025-12-20

**Authors:** Panuwat Yamsakul, Terdsak Yano, Thanaporn Eiamsam-ang

**Affiliations:** School of Veterinary Medicine, Faculty of Veterinary Medicine, Chiang Mai University, Chiang Mai 50200, Thailand; terdsak.yano@cmu.ac.th (T.Y.); thanaporn.e@cmu.ac.th (T.E.-a.)

**Keywords:** non-starch polysaccharide enzymes, pig, digestibility, performance

## Abstract

Non-starch polysaccharides (NSPs) in plant-based swine diets can reduce nutrient availability, and the use of exogenous NSP-degrading enzymes has been proposed as a practical approach to improve digestive utilization. This study examined the effects of a commercial enzyme mixture through both in vitro assessment and an in vivo trial in nursery pigs. The in vitro evaluation of seven commercial diets showed that enzyme supplementation increased dry matter, crude protein, crude fat, and crude fiber digestibility, with the most notable improvements observed in finisher, gestating, and lactating diets. In the in vivo experiment, pigs fed the enzyme-supplemented diet showed higher average daily gain (464 vs. 361 g/d; *p* = 0.07) and an improved feed conversion ratio (1.82 vs. 2.39; *p* = 0.07), together with numerically greater cecal butyrate concentration (2.13 vs. 1.06 mmol/dL; *p* = 0.10) and increased villous height in the jejunum and ileum. Although these responses did not reach statistical significance, they represent consistent trends that align with the in vitro findings and suggest potential benefits in nutrient utilization and gut morphology. Overall, the results indicate that NSP enzyme supplementation may support digestive function under specific dietary conditions, particularly in diets containing moderate to high NSP levels, and provide useful information for its practical application in swine nutrition.

## Simple Summary

Modern pig diets contain natural plant fibers that pigs cannot fully digest, which may limit nutrient absorption and slow growth. Enzyme supplementation has been proposed as a way to help pigs use these nutrients more efficiently. In this study, we examined whether adding a non-starch polysaccharide enzyme mixture could improve the digestibility of several common swine diets. Laboratory tests showed that the enzyme improved nutrient availability in many diet types, particularly those used for finishing pigs and breeding herds. When evaluated in young pigs, the enzyme supplementation showed trends toward better growth performance and healthier intestinal structures. Although these differences were not statistically significant, the overall patterns suggest that enzymes may help pigs obtain more benefit from the same amount of feed. These findings indicate that enzyme supplementation has the potential to support more efficient and sustainable pig production by enhancing nutrient use and promoting digestive health.

## 1. Introduction

Plant-based ingredients constitute the major proportion of modern swine diets, and these materials naturally contain considerable amounts of non-starch polysaccharides (NSP), including β-mannan, xylan, and β-glucan. Most of these compounds are poorly digested by pigs and can increase intestinal digesta viscosity, thereby limiting the efficiency of endogenous digestive enzymes and reducing the availability of nutrients [1]. Elevated viscosity has also been associated with increased fermentation by undesirable microorganisms, a process that may impair gut integrity and compromise growth performance [2]. Because feed cost represents the major expenditure in swine production, improving nutrient utilization through nutritional or technological strategies remains essential for maintaining productivity and long-term sustainability.

The application of exogenous enzymes has therefore received considerable attention as a practical means of enhancing the degradation of fiber-rich components in swine diets. Enzymes such as β-mannanase, xylanase, β-glucanase, and cellulase have been reported to improve nutrient digestibility, modulate microbial populations in the large intestine, and support better growth performance [3,4,5,6]. Previous studies have demonstrated notable benefits, including increases of approximately 15% in daily weight gain when non-starch polysaccharide-degrading enzymes were incorporated into wheat- or barley-based diets [7,8]. However, responses to enzyme supplementation remain inconsistent, with variation arising from differences in diet composition, enzyme characteristics, animal age, and gastrointestinal conditions. As these inconsistencies continue to be reported across studies [9], further evaluation through both controlled in vitro assays and complementary in vivo trials is needed to clarify the circumstances under which such enzymes are most effective.

In this context, the present study aimed to investigate the effects of an exogenous enzyme mixture on nutrient digestibility across multiple swine diet types using in vitro techniques, and to verify these findings through an in vivo nursery pig trial. By integrating laboratory-based digestibility assessments with physiological measurements obtained from live animals, the study sought to provide a more comprehensive understanding of how non-starch polysaccharide degrading enzymes influence feed utilization. This combined approach is expected to contribute to a clearer interpretation of enzyme efficacy under different dietary conditions, thereby supporting informed decision making in practical swine nutrition.

## 2. Materials and Methods

### 2.1. Diets and Exogenous NSP Enzyme

Seven types of commercial swine diets were included in this study: creep, nursery, starter, grower, finisher, gestating, and lactating diets. The ingredient composition and chemical analyses of all diets are shown in Table 1. Each diet was divided into two treatments: a control diet without enzyme supplementation and an enzyme-supplemented diet containing 100 g/ton of Hostazym^®^ X (Huvepharma company limited, Sofia, Bulgaria). This commercial preparation consists of cellulase, α-amylase, protease, and hemicellulase derived from *Trichoderma longibrachiatum*, and was added to evaluate its potential to enhance the degradation of non-starch polysaccharide components under practical feeding conditions.

### 2.2. In Vitro Digestibility Assay

Dry matter and starch digestibility of the seven commercial diets were assessed using a Daisy Incubator system (Ankom Technology, Macedon, NY, USA). For each diet, three independent replicates were analyzed, and blank F57 filter bags were included in every run to allow residue-based correction. A 500 g composite sample of each diet was homogenized, and subsamples were weighed into Ankom F57 bags. For dry matter digestibility, bags were incubated for 0, 12, and 24 h following the manufacturer’s recommendations. All buffer solutions were prepared according to standard Daisy Incubator procedures, and pH was adjusted and verified (±0.05) prior to incubation. The incubation temperature was maintained at 39 ± 0.5 °C to simulate porcine gastrointestinal conditions. Starch digestibility was evaluated following the two-step procedure of Boisen and Fernández (1993) [10], which simulates gastric and small intestinal digestion. Briefly, samples were incubated for 2 h at pH 3.0 in a citrate-phosphate buffer containing pepsin (2000 U/g), followed by 4 h incubation at pH 6.8 in a phosphate buffer containing pancreatin (equivalent to 100 U/g trypsin activity). pH was monitored throughout the assay and adjusted when necessary to maintain the intended digestion environment. After completion of the incubations, all bags (including blanks) were rinsed thoroughly, dried at 130 °C for 2 h, and ashed at 500 °C for 3 h according to AOAC (1980) procedures [11]. Digestibility coefficients were calculated following Boisen and Fernández, using blank-corrected residues, to ensure accurate estimation of nutrient degradation across diet types.

### 2.3. In Vivo Trial in Nursery Pigs (Growth Performance and Digestibility Assay)

The in vivo experiment was conducted using a completely randomized design and was approved by the Ethics Committee of the Faculty of Veterinary Medicine, Chiang Mai University (SO24/2016; 20 April 2016). Ten male starter pigs from a commercial crossbred herd (Large White × Landrace × Duroc), with an initial body weight of 16.71 ± 0.36 kg, were individually housed and randomly assigned to one of two dietary treatments. Five pigs received a standard basal diet (starter diet), whereas the remaining five were fed the same diet supplemented with an NSP enzyme mixture (100 g/ton of Hostazym^®^ X), following the manufacturer’s recommended inclusion rate. Although the commercial preparation contains cellulase, α-amylase, protease, and hemicellulase derived from Trichoderma longibrachiatum, specific activity units were not disclosed due to proprietary formulation. The use of a control group enabled direct comparison under practical feeding conditions.

The sample size was determined prior to the trial based on an a priori power analysis using G*Power version 3.1. Assuming a medium effect size, a significance level of 0.05, and statistical power of 0.80, the minimum required number of animals was calculated as five pigs per treatment. This number was subsequently adopted to comply with ethical considerations regarding animal use and to align with the available individual housing facilities. Randomization was performed using a computer-generated simple random allocation procedure after stratifying pigs by initial body weight to minimize baseline variation. To reduce potential bias, only the researcher responsible for diet preparation was aware of treatment allocation; personnel involved in daily care and sample collection were blinded to dietary treatments, and investigators responsible for laboratory analyses and statistical evaluation were also blinded throughout the study.

All pigs had free access to feed and water during the 14-day experimental period. Growth performance was recorded using initial and final body weight, average daily gain (ADG), and feed conversion ratio (FCR). Chromic oxide (0.5%) was incorporated into the diets seven days prior to fecal collection to allow sufficient adaptation and marker equilibration in the gastrointestinal tract. Fecal samples (100 g per pig) were collected during the final three days of the experiment and stored at −20 °C until analysis. Chromic oxide concentrations in feed and feces were analyzed in duplicate, and recovery values exceeding 95% confirmed the reliability of the marker-based calculations. Apparent total tract digestibility (ATTD) was calculated using standard external-marker equations. At the end of the trial, pigs were humanely euthanized via intravenous thiopental sodium injection, and tissue samples from the duodenum, jejunum, and ileum were collected for histological evaluation.

### 2.4. Laboratory Analyses

To minimize potential measurement bias, all laboratory analyses were conducted under blinded conditions. Tissue samples for histological evaluation were coded by an independent technician prior to fixation, sectioning, and microscopic analysis. The investigator measuring villous height, crypt depth, and related cellular indices therefore had no knowledge of the dietary treatments. For microbiological enumeration, digesta samples were labeled with anonymous identification codes before serial dilution, plating, and colony counting, ensuring that the analyst could not distinguish between control and enzyme-supplemented groups. Digesta viscosity measurements were likewise performed using coded samples, with all readings obtained by a laboratory technician who was not involved in the animal trial and had no access to treatment allocation. These procedures were implemented to reduce observer bias and to maintain analytical consistency across all laboratory assessments.

### 2.5. Digesta Viscosity

Digesta viscosity was measured using a Brookfield Digital Viscometer following standard procedures for swine nutritional studies. Chemical analyses of the feed and fecal samples were performed to support the interpretation of viscosity and digestibility outcomes. Crude protein was analyzed using the Kjeldahl method (AOAC 2001.11), crude fiber according to AOAC 962.09, crude fat following AOAC 920.39, and ash and dry matter according to NFTA 2.1.4 guidelines. Apparent total tract digestibility (ATTD) was calculated using chromic oxide as an external marker, applying the equation described by Fenton and Fenton (1979) [12]. These standardized analytical proce-dures were employed to ensure accuracy, methodological consistency, and compara-bility across all evaluated diet types.

### 2.6. Histological Examination

Intestinal tissue samples from the duodenum, jejunum, and ileum were fixed in a mixed solution of glutaraldehyde and paraformaldehyde, embedded in paraplast, and sectioned at a thickness of 5 μm. Sections were stained with hematoxylin and eosin and examined under light microscopy. For each intestinal segment, villus height was measured from 40 well-oriented villi, while crypt depth and villus-to-crypt ratio were recorded using the same selection criteria. Epithelial cell density was assessed by counting the number of nuclei per defined unit area, and crypt mitotic activity was evaluated from randomly selected crypts. All measurements were performed using the Olympus Imaging Software system, (e.g., cellSens™ Imaging Software, Olympus Corporation, Tokyo, Japan; version 1.16) to ensure consistent calibration and accuracy across samples.

### 2.7. Microbiota and Volatile Fatty Acid Analysis

Digesta samples (1 g) collected from the ileum, cecum, and colon were serially diluted and cultured to enumerate *Escherichia coli*, *Enterobacteriaceae*, and *Lactobacillus* spp. using the spread plate method described by Harrigan and McCance [13]. Volatile fatty acid concentrations were analyzed using gas–liquid chromatography following standard procedures [14]. These analyses were conducted to evaluate potential changes in microbial populations and fermentation activity in response to enzyme supplementation.

### 2.8. Statistical Analysis

All statistical analyses were performed using R software (Version 4.4.1). Because the in vivo trial consisted of two independent dietary treatments without repeated measurements within the same animal, the independent samples t-test was selected as the primary method for comparing treatment means. This approach was considered appropriate for the comparative and applied nature of the study, where the objective was to assess potential treatment differences under practical conditions rather than to build complex mechanistic models.

Before analysis, data were screened for compliance with parametric assumptions. The Shapiro-Wilk test was used to assess normality of residuals, and Levene’s test was applied to confirm homogeneity of variances between groups. When deviations from normality were detected, log transformation was performed. If assumptions were still not met after transformation, non-parametric alternatives were considered.

For all measured variables-including nutrient digestibility, digesta viscosity, intestinal morphology, microbial counts, volatile fatty acids, and growth performance-results are presented as mean ± standard deviation. In addition to *p*-values, effect sizes (Cohen’s d) and their corresponding 95% confidence intervals were calculated to provide information on the magnitude and practical relevance of treatment differences, acknowledging the limited sample size. A significance level of *p* < 0.05 was applied, and tendencies at 0.05 ≤ *p* < 0.10 were reported when biologically meaningful. Additional details regarding the statistical calculations, including effect size estimation and confidence intervals, are provided in the Supplementary Materials. No multiple-comparison correction procedures were required, as only two treatment groups were evaluated.

## 3. Results

### 3.1. In Vitro Nutrient Digestibility

The in vitro digestibility values for dry matter (DM), crude protein (CP), crude fat (EE), and crude fiber (CF) across the seven commercial diets are shown in Table 2. In the creep diet, no significant differences were detected between the control and enzyme-supplemented groups for any of the measured components (*p* > 0.05). The nursery diet showed a similar pattern, with digestibility coefficients remaining comparable between treatments. In contrast, several diet types with higher fiber content-particularly the starter, grower, finisher, gestating, and lactating diets-displayed numerically higher digestibility values when supplemented with the enzyme mixture. Although these improvements did not reach statistical significance for all nutrients, the consistent upward shifts across multiple diet types suggest that the enzyme preparation may support enhanced nutrient release under in vitro conditions, particularly in diets containing greater amounts of non-starch polysaccharides.

Table 2 summarizes the in vitro digestibility of dry matter, crude protein, crude fat, and crude fiber across the seven commercial diets. For the creep diet, no statistically significant differences were detected between the control and enzyme-supplemented groups for any of the analyzed nutrients (*p* > 0.05). In the nursery diet, crude fat digestibility was numerically higher in the enzyme group; however, the difference was not significant.

For the starter diet, crude protein and crude fiber digestibility were lower in the control group, whereas dry matter, crude fat, and ash digestibility were comparable between treatments. In the grower diet, the control group showed lower digestibility of dry matter, crude fat, crude fiber, and ash, while crude protein digestibility remained similar between groups. A consistent numerical pattern was also observed in the finisher diet, where all measured nutrients tended to be lower in the control group, although none reached statistical significance.

For the gestating diet, crude protein, crude fiber, and ash digestibility were reduced in the control group, whereas dry matter and crude fat digestibility were similar between treatments. A similar trend was observed for the lactating diet, in which dry matter, crude protein, and ash digestibility were numerically lower in the control group, while crude fat and crude fiber digestibility did not differ between treatments. Overall, although the differences were not statistically significant, the numerical improvements observed in several diet types are consistent with the expected enzyme activity under higher-NSP dietary conditions.

### 3.2. In Vivo Trial in Nursery Pigs

#### 3.2.1. Growth Performance of Nursery Pigs

Table 3 presents the growth performance of nursery pigs fed either the control diet or the diet supplemented with the NSP enzyme mixture. Although none of the measured parameters differed significantly between treatments (*p* > 0.05), pigs in the enzyme-supplemented group exhibited numerically higher final body weight and greater average daily gain, together with a more favorable feed conversion ratio. These values approached statistical significance (*p* = 0.07) and indicate a consistent trend toward improved growth performance under the conditions of this study.

#### 3.2.2. Volatile Fatty Acid Profiles

Table 4 summarizes the concentrations of volatile fatty acids (VFAs) in the cecum of nursery pigs fed either the control diet or the NSP enzyme–supplemented diet. No statistically significant differences were detected for any individual VFA or for total VFA concentration (*p* > 0.05). Nonetheless, pigs receiving the enzyme-supplemented diet showed a consistent numerical increase in total VFA concentration compared with the control group (*p* = 0.10), indicating a possible shift in hindgut fermentation patterns associated with enzyme inclusion. Although this trend did not reach statistical significance, the directionality of the response may reflect enhanced fermentative activity linked to improved substrate availability.

#### 3.2.3. Digesta Viscosity

**Figure 1:**
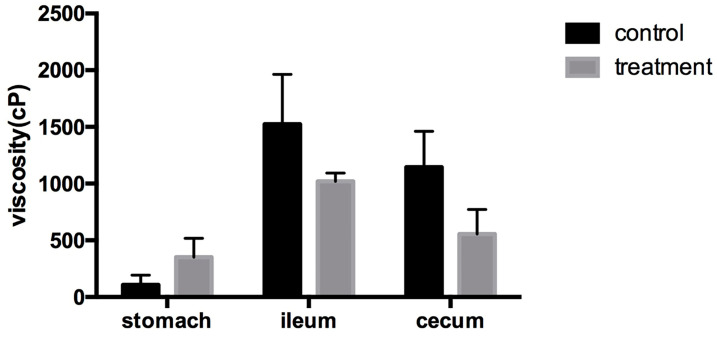
Digesta viscosity in nursery pigs fed the control or NSP enzyme-supplemented diet (n = 5). Values are presented as mean ± SEM.

Figure 1 presents the digesta viscosity values measured in nursery pigs from the control and NSP enzyme–supplemented groups. Although no statistically significant differences were observed between treatments (*p* > 0.05), the enzyme-supplemented pigs showed consistently lower viscosity across all intestinal segments, with more notable numerical reductions in the ileum and cecum. These patterns suggest a potential viscosity-lowering effect of the NSP enzyme mixture, even though the magnitude of change did not reach statistical significance under the conditions of this study.

#### 3.2.4. Microbial Populations

**Figure 2:**
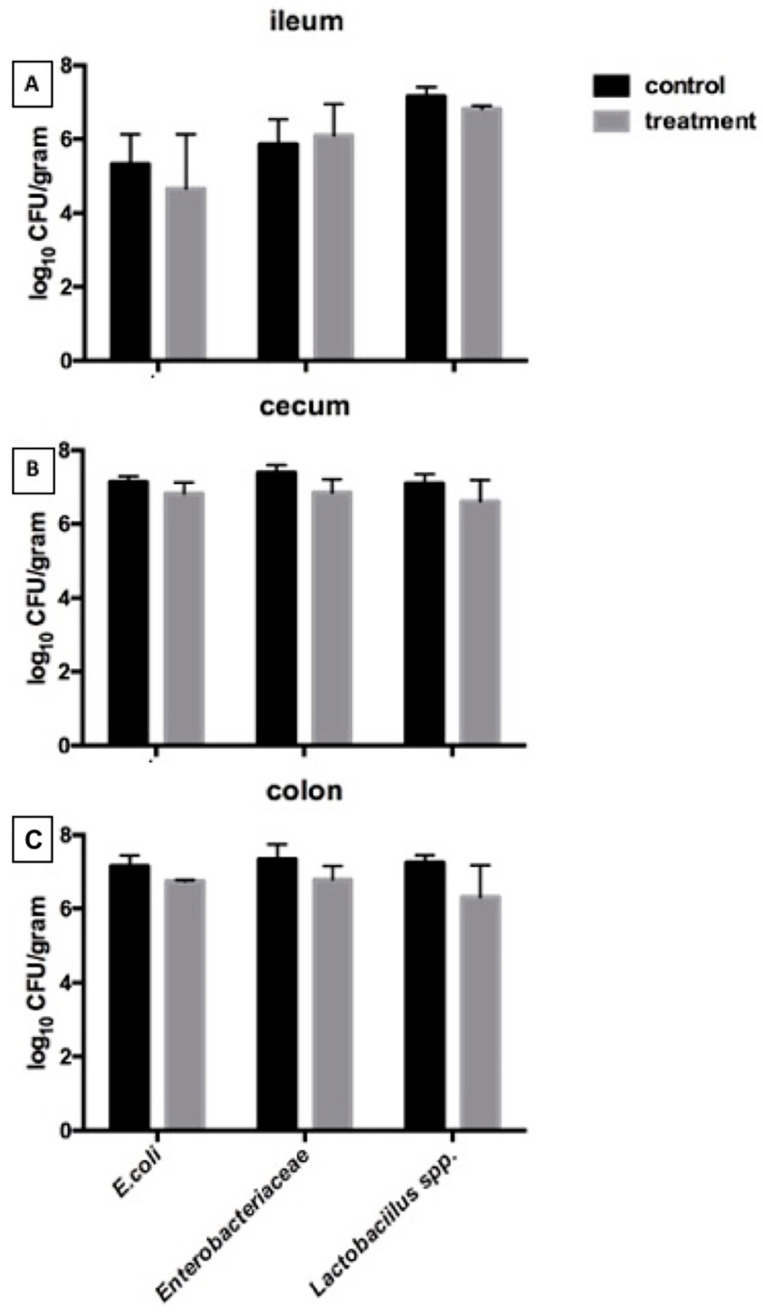
Microbial populations in the (**A**) ileum, (**B**) cecum, and (**C**) colon of nursery pigs fed the control or NSP enzyme-supplemented diet (n = 5). Values are presented as mean ± SEM.

Figure 2 shows the microbial populations in the ileum, cecum, and colon, including *Escherichia coli*, *Enterobacteriaceae*, and *Lactobacillus* spp. No statistically significant differences were observed between the control and NSP enzyme–supplemented groups for any of the evaluated bacterial counts (*p* > 0.05). Although numerical variation was present across intestinal segments, the overall microbial profiles remained comparable between treatments, indicating that the enzyme mixture did not produce measurable changes in these major microbial indicators under the conditions of the present study.

#### 3.2.5. Intestinal Morphology

**Figure 3:**
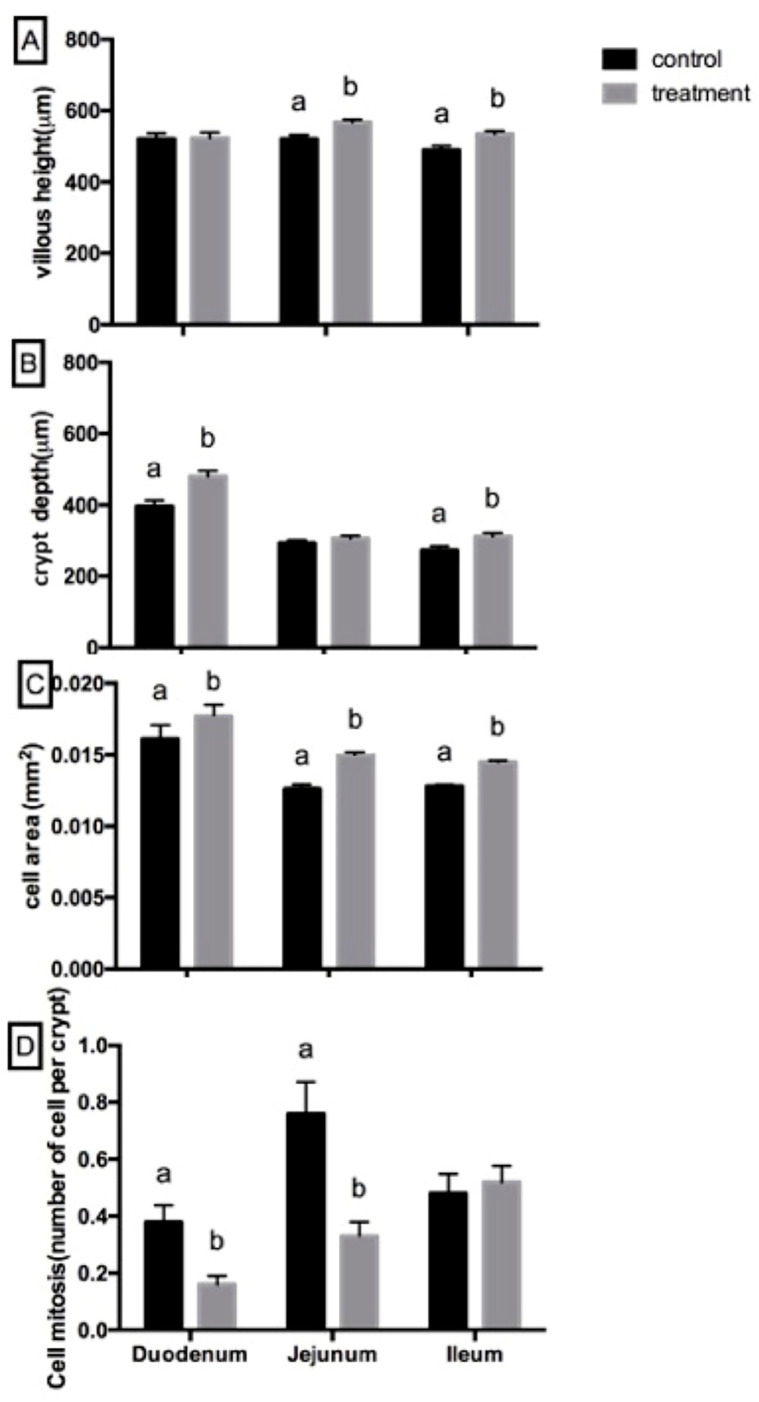
(**A**) Villus height, (**B**) crypt depth, (**C**) epithelial cell area, and (**D**) crypt mitotic counts in the duodenum, jejunum, and ileum of nursery pigs fed either the control diet or the NSP enzyme-supplemented diet (n = 5). Values are presented as mean ± SEM. Different letters (a, b) indicate significant differences between dietary treatments within the same intestinal segment (*p* < 0.05).

Figure 3 illustrates the villous morphology of the duodenum, jejunum, and ileum, including villous height, crypt depth, epithelial cell area, and crypt mitotic counts. Although no statistically significant differences were detected between treatments (*p* > 0.05), the enzyme-supplemented group exhibited a consistent pattern of numerically greater villous height, particularly in the jejunum and ileum. Crypt depth also tended to be higher in the duodenum and ileum of pigs receiving the enzyme-supplemented diet. Epithelial cell area was numerically larger across all intestinal segments in the treatment group, suggesting a potential improvement in epithelial development. Conversely, the number of mitotic cells appeared lower in the duodenum and jejunum of enzyme-fed pigs, while values in the ileum remained comparable between groups. These collective trends may indicate subtle shifts in intestinal structure; however, the magnitude of these changes was modest under the conditions of the present study.

#### 3.2.6. Nutrient Digestibility

**Figure 4:**
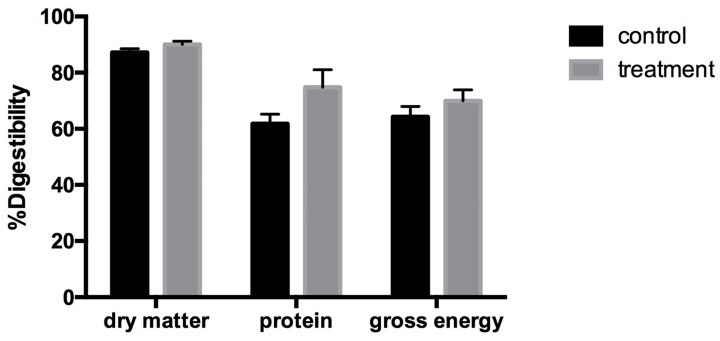
Apparent total tract digestibility (ATTD) of nutrients in feces of nursery pigs fed the control diet or the diet supplemented with an NSP enzyme mixture (n = 5 per group). Values are presented as mean ± SEM.

Figure 4 presents the apparent total tract digestibility (ATTD) of nutrients in nursery pigs fed the control or NSP enzyme-supplemented diets. No statistically significant differences were detected for any of the measured components (*p* > 0.05). Nonetheless, pigs receiving the enzyme-supplemented diet consistently exhibited higher digestibility values for crude protein and gross energy of the trial. Although these differences did not reach statistical significance, the repeated pattern suggests a modest but biologically relevant trend that may reflect improved nutrient utilization under the dietary conditions of this study.

## 4. Discussion

Non-starch polysaccharides (NSPs) exert variable effects on digestive processes depending on their botanical origin, chemical structure, and interactions with other dietary components, particularly in cereal-based ingredients such as wheat and rye [15]. Their ability to increase digesta viscosity can reduce nutrient accessibility by limiting substrate–enzyme contact and impairing endogenous digestive activity [1]. As pigs do not produce sufficient endogenous enzymes to hydrolyze complex NSPs [16], supplementing diets with exogenous NSP-degrading enzymes has been proposed as a practical strategy to enhance nutrient release and digestive efficiency. Consistent with this concept, the present in vitro results demonstrated improved digestibility in several diet types—especially starter, grower, finisher, gestating, and lactating diets, which typically contain higher NSP levels. These findings align with evidence showing that enzyme efficacy depends on both the amount and structure of NSP present in the diet [9,17]. It should also be noted that crude fiber, used to estimate dietary fiber in this study, does not fully represent total dietary fiber, which may influence the precision of fiber-related interpretations. Conversely, the limited response observed in creep and nursery diets suggests that enzyme effects may be reduced when intrinsic NSP content is relatively low.

The in vivo results further demonstrated that although growth performance did not differ statistically between groups, pigs receiving the enzyme-supplemented diet showed consistent tendencies toward higher final body weight, increased average daily gain, and improved feed conversion ratio. These trends align with previous research demonstrating positive but sometimes modest growth responses to NSP-degrading enzymes across different production stages [6,18,19]. However, reduced ileal digestibility was observed, potentially reflecting accelerated digesta transit time, as reported by Chen et al. [20]. Comparable inconsistencies have been documented in pigs fed corn-soybean or wheat-based diets, in which enzyme supplementation resulted in limited or variable responses in growth and digestibility [21,22]. These divergent outcomes highlight the multifactorial nature of enzyme efficacy, which can be influenced by diet formulation, enzyme characteristics, gastrointestinal conditions, animal age, and physiological status. Even so, other reports-including those by Barrera et al. [4] and Emiola et al. [8] have demonstrated clear improvements in finishing pigs receiving NSP enzymes, emphasizing that diet composition is a critical determinant of enzyme effectiveness.

Although digesta viscosity and microbial populations did not differ significantly between treatments, a numerical decrease in viscosity was observed in both the ileum and cecum of pigs receiving enzyme supplementation. Given that arabinoxylans are among the primary drivers of increased digesta viscosity [23], and that xylanase can reduce viscosity by hydrolyzing these polymers [22], the observed trend may still have biological relevance even in the absence of statistical significance. Classical plate-count methods were used to quantify *Escherichia coli, Enterobacteriaceae,* and *Lactobacillus* spp. rather than high-throughput sequencing. This approach was selected based on the specific objectives of the present study and laboratory constraints, and remains widely used for targeted evaluation of viable bacterial groups associated with digestive stability. Previous studies demonstrate that culture-based methods can reliably detect shifts in dominant taxa and can correlate with fermentation responses in pigs fed fiber or enzyme-supplemented diets [24,25,26,27]. The absence of major differences in bacterial counts may reflect the complex microbial ecosystem in the gastrointestinal tract, which is influenced by baseline microbiota composition, feed matrix interactions, animal health status, and environmental factors [28]. Future studies integrating high-throughput microbiome and metabolomic analyses would help clarify relationships among NSP degradation, microbial community structure, and fermentation outcomes [29,30].

Although the differences in cecal volatile fatty acids (VFAs) were not statistically significant, pigs receiving enzyme supplementation tended to show higher total VFA concentrations. This pattern is consistent with the mechanistic expectation that hydrolysis of NSPs releases soluble carbohydrates and oligosaccharides that serve as fermentable substrates for hindgut microbiota. Previous studies have demonstrated that NSP degradation mediated by xylanase or cellulase can enhance fermentation of arabinoxylans and β-glucans, resulting in increased production of acetate, propionate, and butyrate in pigs [31]. These VFAs contribute to epithelial energy supply, mucosal integrity, and modulation of gut motility. Therefore, even modest increases in VFA concentrations may indicate enhanced fermentation and a more functionally active microbial environment. The lack of statistical significance in the present study is likely associated with the small sample size and moderate NSP content of typical commercial diets, yet the observed patterns remain consistent with reports of enzyme-modulated fermentation responses in pigs.

Morphological evaluation of the small intestine revealed tendencies toward increased villous height, deeper crypts, and larger epithelial cell area in pigs receiving enzyme supplementation, particularly in the jejunum and ileum. Such changes may support improved absorptive capacity. Previous studies have reported that NSPs can influence epithelial turnover and gut morphology [32], whereas NSP-degrading enzymes may alleviate NSP-associated constraints and enhance mucosal integrity [33]. The observed reduction in cell mitosis in the duodenum and jejunum may indicate more stable epithelial turnover and reduced proliferative stress-patterns similar to those reported in studies showing improved intestinal health following exogenous enzyme supplementation [16,34,35]. These physiological responses may partly explain the favorable trends in growth performance despite the absence of statistically significant differences.

Taken together, the findings of this study support the hypothesis that NSP-degrading enzymes can enhance nutrient utilization and modulate digestive physiology, particularly when diets contain substantial NSP levels. Although most outcomes appeared as trends rather than statistically significant differences, this pattern likely reflects the limited sample size and the use of conventional raw materials with only moderate NSP content. Nevertheless, the consistent tendencies observed in both the in vitro and in vivo evaluations indicate that NSP enzyme supplementation can contribute to improved nutrient release and digestive responses under practical feeding conditions. More pronounced effects may be expected in future studies using higher-fiber or alternative raw materials with more complex NSP profiles. Further research should therefore include detailed characterization of NSP fractions, evaluation of enzyme-substrate interactions in diverse diet matrices, and integration of microbial and metabolite analyses to elucidate underlying mechanisms. Such information will support refinement of feeding strategies and enhance the practical application of NSP-degrading enzymes in modern swine production. Moreover, another factor that may help explain the clearer responses observed in the in vitro assay compared with the more modest changes in vivo is the potential difference in enzyme stability along the gastrointestinal tract. According to information provided by the manufacturer, the commercial enzyme preparation (Hostazym^®^ X) is formulated as a stabilized granulate to maintain activity during feed handling and digestion. However, the degree to which the enzymes remain active until reaching specific intestinal sites cannot be fully assured. Partial deactivation during gastric transit or feed processing may therefore contribute to the attenuated responses observed in vivo. This consideration highlights the importance of evaluating not only enzyme inclusion levels but also enzyme stability when interpreting efficacy under practical feeding conditions.

Finally, we acknowledge the limitation that independent t-test may not fully capture complex variation in multi-parameter datasets. However, because the present experiment involved only two independent dietary treatments without repeated measures within animals, the independent t-test was considered appropriate. To strengthen interpretation, effect sizes and confidence intervals were reported as recommended by the reviewer. Future studies with larger sample sizes and more complex designs should consider mixed models to improve statistical power and account for hierarchical variation.

## 5. Conclusions

The present study indicates that supplementation with non-starch polysaccharide degrading enzymes may help support improved nutrient utilization in swine diets, especially those containing moderate to high levels of fiber. Although most in vivo responses did not reach statistical significance, consistent numerical tendencies in growth performance, apparent nutrient digestibility, volatile fatty acid production, and intestinal morphology suggest that the enzyme mixture may contribute to more favorable digestive conditions under certain dietary and physiological contexts. These modest responses likely reflect the small sample size and the use of conventional feed ingredients with only moderate NSP content, which may have limited the potential magnitude of enzyme effects. The effectiveness of NSP-degrading enzymes is known to depend on multiple factors, including the type and concentration of dietary NSPs, animal age and physiological status, and the digestive environment within the gastrointestinal tract. Future studies incorporating larger experimental groups, diets formulated with alternative or higher-NSP raw materials, and detailed characterization of NSP fractions will be essential for clarifying enzyme-substrate interactions and improving mechanistic understanding. Such efforts will help refine feeding strategies and enhance the practical application of NSP enzymes in modern swine production systems.

## Figures and Tables

**Table 1:** Ingredient composition and chemical analysis of the experimental diets.

Items	Type of Diets						
	Creep	Nursery	Starter	Grower	Finisher	Gestating	Lactating
Ingredients, %							
Broken rice	43.23	51.46	16.78	14.70	9.95	38.40	43.52
Corn	N	N	30.70	30.88	30.86	N	N
Soy bean oil	N	N	N	N	N	N	2.96
Full fat soy bean	21.61	25.49	3.83	N	N	N	16.32
Soy bean meal	3.93	1.96	24.47	24.65	16.92	16.74	13.35
Fish meal	N	N	0.47	0.47	0.99	N	N
Whey	9.81	4.90	N	N	N	N	N
Rice barn	N	N	12.95	14.80	29.85	30.02	19.77
Snack from cassava	N	N	5.94	4.92	6.25	N	N
Minor ingredients	21.42	16.19	4.86	9.58	5.18	14.84	4.08
Analyzed composition							
DM, %	92.63	91.08	92.95	92.48	91.98	91.24	91.82
CP, %	26.18	23.69	23.43	20.87	19.93	19.75	23.25
Fat, %	9.18	8.79	6.90	6.63	7.94	5.40	7.83
CF, %	3.00	3.79	5.66	7.52	7.71	9.86	5.08

Notes: N = Not included in the feed formulation; DM = Dry matter; CP = Crude protein; Fat = Crude fat; CF = Crude fiber. Minor ingredients represent the remaining proportion of the diet after major ingredients were accounted for (to total 100%) and consisted of monocalcium phosphate, calcium carbonate, sodium chloride, and vitamin–mineral premix.

**Table 2:** In vitro digestibility (%) of dry matter (DM), crude protein (CP), crude fat (EE), and crude fiber (CF) in seven commercial swine diets with or without NSP enzyme supplementation.

Group	Percentage of Digestibility of Each Nutrient				
	Dry Matter (DM)	Crude Protein (CP)	Crude Fat (Fat)	Crude Fiber (CF)	Ash (Ash)
Creep diet					
control	51.83 ± 1.73	47.57 ± 4.03	30.42 ± 1.30	41.26 ± 4.11	24.51 ± 5.52
treatment	49.90 ± 2.34	46.23 ± 1.06	29.26 ± 3.59	44.88 ± 1.97	30.57 ± 3.41
Nursery diet					
control	40.99 ± 5.50	50.46 ± 1.17	29.63 ± 3.02 **^b^**	42.62 ± 0.62	20.57 ± 4.16
treatment	40.04 ± 1.92	49.94 ± 1.38	33.92 ± 1.72 **^a^**	43.94 ± 4.16	20.33 ± 4.20
Starter diet					
control	51.38 ± 2.50	48.31 ± 1.10 **^b^**	36.98 ± 3.48	52.69 ± 3.10 **^b^**	23.37 ± 4.60 **^b^**
treatment	52.64 ± 3.70	51.01 ± 2.08 **^a^**	35.33 ± 7.51	57.04 ± 4.11 **^a^**	24.04 ± 2.53 **^a^**
Grower diet					
control	49.50 ± 2.04 **^b^**	43.58 ± 1.50	23.34 ± 1.17 **^b^**	49.55 ± 3.18 **^b^**	21.69 ± 2.64 **^b^**
treatment	50.74 ± 0.79 **^a^**	42.20 ± 0.20	28.58 ± 2.76 **^a^**	52.63 ± 1.58 **^a^**	27.52 ± 1.52 **^a^**
Finisher diet					
control	60.20 ± 1.32 **^b^**	56.46 ± 0.25 **^b^**	29.25 ± 2.36 **^b^**	40.88 ± 2.86 **^b^**	21.69 ± 2.64 **^b^**
treatment	69.12 ± 2.11 **^a^**	58.46 ± 0.65 **^a^**	32.59 ± 2.36 **^a^**	43.98 ± 1.69 **^a^**	27.52 ± 1.52 **^a^**
Gestating diet					
control	48.56 ± 0.46	54.14 ± 1.07 **^b^**	32.31 ± 3.54	54.25 ± 1.27 **^b^**	25.56 ± 1.18 **^b^**
treatment	47.92 ± 0.15	56.45 ± 1.06 **^a^**	35.05 ± 1.41	57.28 ± 1.77 **^a^**	28.33 ± 2.59 **^a^**
Lactating diet					
control	51.45 ± 1.15 **^b^**	63.75 ± 1.19 **^b^**	35.28 ± 3.02	42.82 ± 1.65	25.10 ± 0.15 **^b^**
treatment	54.08 ± 0.37 **^a^**	67.37 ± 1.47 **^a^**	39.57 ± 2.12	47.86 ± 3.02	28.86 ± 1.61 **^a^**

Different superscripts within the same row indicate significant differences between treatments (*p* < 0.05). Values are presented as mean ± standard error of the mean (SEM) based on three independent replicate incubations.

**Table 3:** Growth performance of nursery pigs fed the control diet or the NSP enzyme–supplemented diet (n = 5).

Indices of Growth Performance	Control Group	Treatment Group	*p*-Value
Initial weight (kg)	16.66 ± 0.94	16.76 ± 0.70	0.93
The weight at 1 week (kg)	19.04 ± 1.14	19.98 ± 0.68	0.49
Final weight (kg)	21.72 ± 1.30	23.26 ± 0.71	0.32
Body weight gain (kg)	5.06 ± 0.68	6.50 ± 0.21	0.07 *
Total feed intake (g)	11,341 ± 656.87	11,770 ± 175.50	0.54
Average daily feed intake (g)	809.10 ± 104.91	840.71 ± 28.03	0.54
Average daily gain (ADG) (g/d)	361.43 ± 46.92	464.28 ± 12.54	0.07 *
Feed conversion ratio (FCR)	2.39 ± 0.28	1.82 ± 0.06	0.07 *

Values represent mean ± standard error of the mean (SEM). * Indicates a statistical trend (0.05 ≤ *p* < 0.10).

**Table 4:** Cecal volatile fatty acid concentrations in nursery pigs fed the control or NSP enzyme–supplemented diet (n = 5).

Volatile Fatty Acid	Control Group	Treatment Group	*p*-Value
Acetate (C2)	13.46 ± 2.25	23.45 ± 8.44	0.31
Propionate (C3)	3.15 ± 1.15	4.92 ± 1.74	0.37
Isobutyrate (Iso-C4)	0.04 ± 0.008	0.05 ± 0.01	0.61
Butyrate (C4)	1.06 ± 0.12	2.13 ± 0.74	0.10 *
Valerate (C5)	0.19 ± 0.03	0.34 ± 0.13	0.33

Values are expressed as mmol/dL and presented as least squares means ± SEM. * Indicates a statistical trend (*p* < 0.10).

## Data Availability

The data supporting the findings of this study are available from the corresponding author upon reasonable request and were generated and used with permission from Chiang Mai University, Thailand. Supplementary information on the commercial enzyme product used in this study (Hostazym X^®^), including registration and regulatory safety assessment, was obtained from publicly available sources. These sources were used solely to provide background information on the enzyme product and did not influence data collection or analysis.

## Supplementary Materials

The following supporting information can be downloaded at: https://www.mdpi.com/article/10.3390/biology15010013/s1 for Table in excel: Raw experimental data provided as an Excel file, including digesta viscosity, feed intake, body weight, growth performance, volatile fatty acid concentrations, intestinal morphology, microbial counts (*Escherichia coli*, *Lactobacillus* spp., and total microbiota), and in vitro and in vivo nutrient digestibility measurements, together with calculated digestibility results.

## Abbreviations

The following abbreviations are used in this manuscript:

NSPs	Non-Starch Polysaccharides
ADG	Average Daily Gain
FCR	Feed Conversion Ratio
DM	Dry Matter
CP	Crud Protein
Fat	Crud Fat
CF	Crud Fiber
